# Modified STOP-Bang Tool for Stratifying Obstructive Sleep Apnea Risk in Adolescent Children

**DOI:** 10.1371/journal.pone.0142242

**Published:** 2015-11-18

**Authors:** Daniel Combs, James L. Goodwin, Stuart F. Quan, Wayne J. Morgan, Sairam Parthasarathy

**Affiliations:** 1 Department of Pediatrics, University of Arizona, Tucson, AZ, United States of America; 2 Arizona Respiratory Center, University of Arizona, Tucson, AZ, United States of America; 3 Department of Medicine, University of Arizona, Tucson, AZ, United States of America; 4 Division of Sleep and Circadian Disorders, Brigham and Women’s Hospital and Division of Sleep Medicine, Harvard Medical School, Boston, MA, United States of America; 5 Center for Sleep Disorders and Division of Pulmonary, Allergy, Critical Care and Sleep Medicine, University of Arizona, Tucson, AZ, United States of America; National Institute for Viral Disease Control and Prevention, CDC, China, CHINA

## Abstract

**Purpose:**

Obstructive sleep apnea (OSA) is prevalent in children and diagnostic polysomnography is costly and not readily available in all areas. We developed a pediatric modification of a commonly used adult clinical prediction tool for stratifying the risk of OSA and the need for polysomnography.

**Methods:**

A total of 312 children (age 9–17 years) from phase 2 of the Tucson Children’s Assessment of Sleep Apnea cohort study, with complete anthropomorphic data, parent questionnaires, and home polysomnograms were included. An adolescent modification of STOP-Bang (teen STOP-Bang) was developed and included snoring, tired, observed apnea, blood pressure ≥ 95th percentile, BMI > 95th percentile, academic problems, neck circumference >95th percentile for age, and male gender. An apnea-hypopnea index ≥ 1.5 events/hour was considered diagnostic of OSA.

**Results:**

Receiver Operator Characteristic (ROC) curves for parent-reported STOP-Bang scores were generated for teenage and pre-teen children. A STOP-Bang score of < 3 in teenagers was associated with a negative predictive value of 0.96. ROC curves were also generated based upon child-reported sexual maturity rating (SMR; n = 291). The ability of teen STOP-Bang to discriminate the presence or absence of OSA as measured by the AUC for children with SMR ≥ 4 (0.83; 95%CI 0.71–0.95) was better than children with SMR < 4 (0.63; 95%CI 0.46–0.81; p = 0.048).

**Conclusions:**

In community dwelling adolescents, teen STOP-Bang may be useful in stratifying the risk of OSA.

## Introduction

In children and adolescents, obstructive sleep apnea (OSA) has a prevalence of 1.2–5.7% [1] and has been associated with behavioral, neurocognitive, learning and cardiovascular problems [2]. Diagnosis and management of OSA in children and adolescents can mitigate the adverse effects of OSA [3–5]. Unfortunately, children are infrequently screened in the primary care setting despite the potential benefits of early identification and treatment of OSA [6]. Additionally, pediatric polysomnography is costly and not widely available in areas without specialized pediatric centers. The issues regarding the under-diagnosis of OSA are further aggravated by the obesity epidemic and likely rising prevalence of OSA in children [7]. The validation of a negative predictive tool would allow appropriate selection of children for polysomnography in limited resource settings.

Several tools for the prediction of OSA in children have been developed. The Pediatric Sleep Questionnaire subscale for sleep-related breathing disorders, consists of 22 questions and was designed primarily as a research instrument rather than a clinical prediction tool aimed at reducing the need for polysomnography [8]. Additionally, it is a lengthy questionnaire and may not be easily administered in a busy “real-world” primary care setting. An alternative tool is the OSA-18 quality of life questionnaire, but this instrument does not accurately exclude moderate-to-severe OSA in children [9].

In adults, an instrument derived from eight easily administered questions called the STOP-Bang questionnaire has been shown to stratify the risk of OSA, with a negative predictive value of 93% for moderate OSA and 96% for severe OSA [10]. This tool was originally developed to predict the risk of OSA during pre-operative evaluation in adults [11]. We hypothesized that a modified version of the STOP-Bang tool may be useful as a clinical prediction tool to determine the need for polysomnography in children, particularly adolescents, who may have a more adult-like phenotype of OSA. In this study, we developed a modified STOP-Bang tool (teen STOP-Bang) for adolescent children and assessed the ability of this tool to stratify the risk of OSA in community-dwelling adolescents. This clinical prediction tool may be useful in a primary care setting to determine the need for polysomnography and further evaluation for OSA in adolescents.

## Methods

### Study Population

This study is obtained from a systematic analysis of phase 2 of the Tucson Children’s Assessment of Sleep Apnea (TuCASA) cohort study. A detailed description of the TuCASA study has been previously described [12]. In brief, Caucasian and Hispanic children were recruited from the Tucson Unified School District, a large district representative of the Tucson population. A total of 503 children and their parents were consented and participated in phase 1 of the study. Approximately 5 years later (mean 4.7 years), 348 children participated in phase 2 of the study. We selected Phase 2 for the current analysis because both pre-teens and teenagers are adequately represented. The TuCASA study was approved by the University of Arizona Institutional Review Board (IRB approval # 0300000227) and the Tucson Unified School District Research Committee. Prior to undergoing study-related procedures, written informed consent was obtained from the parents and minor’s assent was also obtained. Subjects underwent home polysomnograms, and both children and their parents filled out sleep questionnaires pertaining to the children’s sleep habits. Anthropomorphic measurements were also obtained during the home visit.

### Polysomnography

A single, unattended overnight polysomnogram was obtained with the Compumedics PS-2 system (Abbotsford, Victoria, Australia). The following signals were acquired as part of the TuCASA montage: C3/A2 and C4/A1 electroencephalogram, right and left electrooculograms, a bipolar submental electromyogram, thoracic and abdominal displacement (inductive plethysmography), airflow (nasal/oral thermistor), nasal pressure cannula, finger pulse oximetry, electrocardiography (single bipolar lead), snoring microphone, body position (Hg gauge sensor), and ambient light levels [12]. A previous study examined the characteristics of the Compumedics PS-2 system in the TuCASA cohort. The initial pass rate was 91%, which improved to 97% when 9 children who failed on the first night of recording completed a second study which was acceptable. The Compumedics system was validated by performing conventional polysomnography in a subset of subjects, which showed good correlation between the two studies [12].

Scoring of sleep was performed by a single registered polysomnographic technologist using standard criteria[13]. Apneas were scored if the amplitude of the thermistor airflow decreased below at least 25% of the amplitude of baseline breathing, and lasted for more than 6 seconds or 2 breath cycles [2, 12, 14]. Hypopneas were designated if the amplitude of any respiratory signal decreased below 70% of the amplitude of baseline, was associated with a 3% oxygen desaturation, and the thermistor signal did not meet the criterion for apnea [2, 12]. Central events were marked if no displacement was noted on both the chest and abdominal inductance channels. However, central events that occurred after movement were not included. The Apnea Hypopnea Index (AHI) was defined as the number of apneas and hypopneas per hour of total sleep time. For data analysis, an AHI ≥1.5 was considered to indicate OSA, based on prior research identifying this threshold as significantly abnormal [15].

### Anthropometry

All anthropomorphic measures were obtained during the home visit, the evening of the subject’s polysomnograms. Weight was obtained as the average of 3 measurements, to the nearest 0.1 Kg. Height was obtained using a folding ruler on a level surface after removal of shoes, and the head in the Frankfort plane. The average of three measurements to the nearest 0.1 cm was used for data analysis. Body mass index (BMI) was calculated as weight in kilograms divided by height in meters squared. BMI percentiles for age and gender were calculated using Center for Disease Control growth charts [16]. To obtain blood pressures, arm circumference was first measured, and then an appropriate sized blood pressure cuff was selected. Seated blood pressures were obtained in triplicate. The average of the 2^nd^ and 3^rd^ measurements was used for data analysis. Blood pressure percentiles based on height, age, and gender were determined using CDC data [17]. The method of Lohman, Roche and Martorel was used to measure the circumference of the neck [18]. The participant sat upright with the head in the Frankfort Horizontal Plane and an inelastic tape was applied around the neck just below the laryngeal prominence. Then the neck circumference measurement was made perpendicular to the long axis of the neck (which was not necessarily in the horizontal plane). The pressure on the tape was kept to the minimum required to maintain skin contact. The neck circumference measurement was completed in less than 5 seconds, to avoid participant discomfort and was measured to the nearest 1/2 cm and rounded up. Neck circumference percentiles for age and gender were determined through use of a reference data set of healthy weight children [19]. Self-reported sexual maturity ratings (SMR) were also collected using a validated pubertal self-assessment questionnaire [20] without parental supervision that consists of simple line drawings based on photographs of the Tanner and Marshall standards [21, 22].

### Modified STOP-Bang questionnaire

The following were included as the teen STOP-Bang tool: Snoring (How often does your child snore loudly?), Tired (Is your child sleepy during the daytime?), Observed apnea (Does your child stop breathing during sleep?), systolic or diastolic blood pressure greater than or equal to 95^th^ percentile for height and age, BMI greater than 95^th^ percentile for age, Academic problems (Does your child have learning problems?), Neck circumference greater than 95^th^ percentile for age, and male Gender. Answers to the questions had choices that included—don’t know, never, rarely, occasionally, frequently or almost always. The responses were collapsed into a positive or negative response in the following manner. Frequently or almost always was considered as a positive response and responses of don’t know, never, rarely, or occasionally were all considered as a negative response. The number of positive responses was calculated as the subject’s teen STOP-bang score. In the teen STOP-Bang questionnaire the question regarding academic problems was inserted in the place of age greater than 50 years that was used the original version of the STOP-Bang questionnaire for use in adults, because learning problems have been shown to be a symptom of sleep-disordered breathing in children [23].

### Data Analysis

Statistical analysis was performed using SPSS Version 22 (IBM, Armonk, NY). Data was assessed for a normal distribution using a one sample Kolmogorov–Smirnov test. Receiver operating characteristic (ROC) curves were generated based on teen STOP-Bang scores. Separate ROC curves were generated and compared based on age as well as SMR status. This was done to test the hypothesis that the teen STOP-Bang would be more accurate in adolescent children. Both age and SMR status were used as age is a more useful clinical measure, while SMR is a more accurate measure of progression through puberty. An SMR of 4 was used as a split point to differentiate children in earlier vs. later phase of puberty. This SMR correlates with an average age of approximately 13–14 years [21]. Sensitivity, specificity, negative predictive value, positive predictive value, and likelihood ratios were calculated by generating 2x2 contingency tables. Prevalence-adjusted likelihood ratios were also calculated, as the prevalence of OSA in the cohort was 0.08 and non-adjusted likelihood ratios may be misleading when sample prevalence is substantially different from 0.50 [24, 25]. Wilcoxon-signed rank tests were used to determine significance for comparisons in paired data, and Mann-Whitney U tests were used for un-paired data, as the teen STOP-Bang scores were not normally distributed. A p-value of less than 0.05 was considered significant.

## Results

Of the 348 children in the second phase of the TuCASA study, 312 had complete anthropomorphic data, complete questionnaires and acceptable polysomnograms, and were included in data analysis. Demographic data is described in **Table 1**. Twenty-five children were found to have OSA defined as AHI ≥ 1.5/hour measured by polysomnography at the participants’ home. OSA was mild (AHI between 1.5 and 5) in all but five cases, with one case of severe OSA (AHI of 31).

**Table 1:** Demographics of participants.

	No OSA AHI <1.5/hour	OSA AHI ≥ 1.5/hour	P value
	(n = 287)	(n = 25)	
**Ethnicity**			0.43
Caucasian	184 (64%)	18 (72%)	
Hispanic	103 (36%)	7 (28%)	
**Age, years**	13.3 (9.9–17.6)	13.6 (10.5–17.4)	0.48
**Gender**			.07
**Male**	141 (49.1%)	17 (68%)	
**Female**	146 (50.9%)	8 (32%)	
**BMI Percentile**	67.4 (35.2–89.7)	94.3 (81.6–97.8)	<0.001
**Neck percentile**	95 (75–95)	95 (82.5–95)	0.9
**Teen STOP-Bang score**			
Parental report	1 (1, 2)	3 (1.5, 3)	<0.001
Self report	1 (1, 2)	2 (1.5, 3)	0.003
**AHI**	0.2 (0.1, 0.4)	2.2 (1.8, 3.4)	<0.001
**ODI**	0.3 (0.1, 0.6)	2.4 (1.9, 3.5)	<0.001
**Arousal Index**	5.9 (4.6, 7.3)	7.4 (5.3, 10.9)	<0.001

OSA = obstructive sleep apnea; AHI = apnea-hypopnea index; BMI = body mass index; ODI = 3% oxygen desaturation index; Arousal index calculated as total number arousals divided by hours of total sleep time. Data is presented as proportion or median, 25^th^ and 75^th^ quartiles.

**Fig 1:**
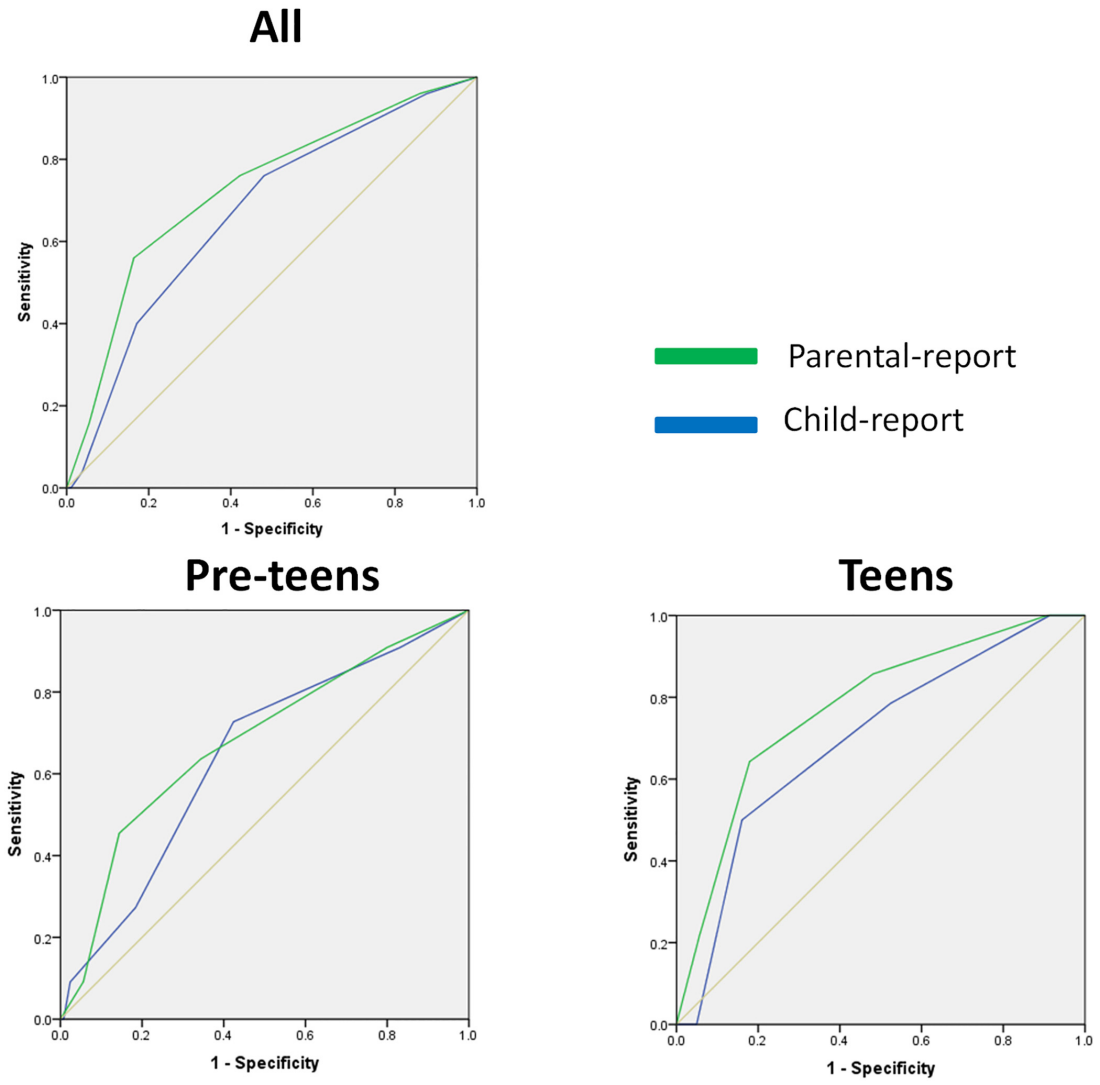
Receiver operating characteristics of teen STOP-Bang. Receiver operating characteristics (ROC) for modified STOP-Bang scores that were child-reported (blue lines) or parent-reported (green line) in discriminating presence of obstructive sleep apnea (defined as an apnea-hypopnea index ≥ 1.5 / hour). The ROC area under the curve for all (top left), pre-teens (bottom left) and teens (bottom right) are shown.

Receiver operating characteristic (ROC) curves were generated for both parent-reported as well as child-reported teen STOP-Bang scores (**Fig 1**). ROCs were also generated separately for pre-teen (9–12 yr) and teenage children (≥13 yr). For teenage children, the area under the curve (AUC) for the ROC was 0.77 (95% confidence interval [CI]; 0.65, 0.90) for parent-reported score, and 0.70 (95% CI; 0.56, 0.84) for child-reported score. For pre-teen children, the AUC was 0.68 (0.51, 0.85) for parent-reported score and 0.64 (0.48, 0.81) for child-reported score. The p-value for the difference between the AUC of ROCs parent-reported score for pre-teens and teenagers was 0.42. A teen STOP-Bang score of ≥ 3 in teenagers was associated with a sensitivity of 64%, specificity of 82%, positive predictive value of 0.24 and negative predictive value of 0.96. Sensitivity and specificity were higher in teenage children when compared to pre-teen children. Test characteristics for different teen STOP-Bang scores for are listed in **Table 2**.

**Table 2:** Test Characteristics of teen STOP-Bang Scores with sensitivity analysis for various thresholds.

Variable	Sensitivity	Specificity	PPV	NPV	LR+	LR-	LR+[P]	LR-[P]
**Teen STOP-Bang score of ≥3**								
All	**0.56**(0.35–0.75)	**0.84**(0.79–0.88)	**0.20**(0.15–0.24)	**0.96**(0.92–0.98)	**3.4**(2.2–5.3)	**0.5**(0.3–0.8)	**0.3**(0.2–0.5)	**0.05**(0.03–0.08)
Teens	**0.64**(0.36–0.86)	**0.82**(0.75–0.88)	**0.24**(0.12–0.41)	**0.96**(0.92–0.98)	**3.6**(2.2–6.0)	**0.4**(0.2–0.9)	**0.3**(0.2–0.6)	**0.04**(0.02–0.09)
Preteens	**0.45**(0.18–0.75)	**0.86**(0.78–0.91)	**0.21**(0.08–0.44)	**0.95**(0.88–0.98)	**3.2**(1.5–6.9)	**0.6**(0.4–1.1)	**0.3**(0.1–0.6)	**0.06**(0.03–0.12)
**Teen STOP-Bang score of ≥2**								
All	**0.76**(0.54–0.89)	**0.58**(0.52–0.64)	**0.13**(0.09–0.21)	**0.97**(0.92–0.99)	**1.8(**1.4–2.3)	**0.4**(0.2–0.8)	**0.2**(0.1–0.2	**0.04**(0.02–0.08)
Teens	**0.86**(0.56–0.97)	**0.52**(0.44–0.60)	**0.13**(0.07–0.23)	**0.98**(0.91–0.996)	**1.8** (1.4–2.3)	**0.3** (0.1–1.0)	**0.2** (0.1–0.3)	**0.02**(0.006–0.09)
Preteens	**0.64**(0.32–0.88)	**0.66**(0.57–0.74)	**0.14**(0.06–0.27)	**0.95**(0.88–0.98)	**1.8** (1.1–3.1)	**0.6**(0.3–1.2)	**0.2**(0.1–0.3)	**0.05**(0.02–0.13)
**Teen STOP-Bang score of ≥4**								
All	**0.16**(0.05–0.37)	**0.94**(0.91–0.97)	**0.20**(0.07–0.44)	**0.93**(0.89–0.95)	**2.9**(1.0–7.9)	**0.9**(0.7–11)	**0.3**(0.1–0.6)	**0.08**(0.0.05–0.12)
Teens	**0.21**(0.06–0.51)	**0.94**(0.89–0.97)	**0.25**(0.07–0.57)	**0.93**(0.88–0.96)	**3.8**(1.1–12.6)	**0.8**(0.6–1.1)	**0.3**(0.1–0.9)	**0.07**(0.04–0.13)
Preteens	**0.09**(0.005–0.43)	**0.94**(0.88–0.98)	**0.13**(0.007–0.53)	**0.92**(0.86–0.96)	**1.6**(0.2–12.0)	**1.0**(0.8–1.2)	**0.1**(0.02–0.9)	**0.08**(0.05–0.15)

Test performance of teen STOP-Bang with various score cut-off thresholds. A teen STOP-Bang score of ≥3 has the best test characteristics. Mean values are in bold font, and 95% confidence intervals are within parenthesis; PPV = Positive Predictive Value; NPV = Negative Predictive Value; LR+ = Likelihood ration of a positive test; LR- = Likelihood ratio of a negative test; LR+[P] = Likelihood ratio of a positive test weighted for prevalence; LR-[P] = Likelihood ratio of a negative test weighted for prevalence. In general an LR+[P] of greater than 5 and LR[–]P of less than 0.2 are considered as indicative of good test characteristics.

Sensitivity analysis was performed for AHI thresholds of 1 and 3 events per hour. For both thresholds, the teen STOP-Bang AUC was inferior. The AUC for a threshold of 1 was 0.68 (95% CI; 0.60–0.77), with 46 children with an AHI ≥ 1. The AUC for a threshold of 3 was 0.73 (95% CI; 0.53–0.92), with 7 children with an AHI ≥ 3. We also performed sensitivity analysis evaluating different BMI percentile cut points of 90th and 99th percentile, but found that these did not improve test characteristics.

To determine whether pubertal status influences the test characteristics of the teen STOP-Bang, we generated ROC curves stratified by self-reported sexual maturity rating (SMR threshold of 4). For children with a SMR ≥ 4, the parent-reported teen STOP-Bang AUC was 0.83 (95%CI; 0.71, 0.95). For children with a SMR < 4, the parent-reported teen STOP-Bang AUC was 0.63 (95% CI; 0.46–0.81). The AUC for the ROCs stratified by sexual maturity were significantly different (p = 0.048).

The ability of parent-reported scores to predict OSA was compared to that of child-reported scores. For teenage children, there was no difference between the parent- and child-reported scores (p = 0.97). For pre-teen children, a significant difference was seen between parent and child-reported scores (p = 0.028; **Table 3**).

**Table 3:** Parent- versus self-reported Teen STOP-Bang scores.

	Sample size	Parent or caregiver reported score	Child-reported score	P value
**All**	312	1 (1, 2)	2 (1, 2)	0.17
**Preteen**	136	1 (1, 2)	1 (1, 2)	0.03
**Teen**	176	2 (1, 2)	2 (1, 2)	0.97

Data is presented as proportion or median, 25^th^ and 75^th^ quartiles. Child and parent-reported scores are similar based on the median and inter-quartile range, however, a significant difference was seen in pre-teens because individual scores did not match well between parent and child dyads.

Finally, we evaluated the necessity of including neck circumference as a measure in teen STOP-Bang. While BMI and blood pressure are routinely measured in the pediatric office, neck circumference is not a standard measure, and inclusion of this variable may limit implementation into clinical practice. We generated a new score using all teen STOP-Bang variables except neck circumference (teen STOP-Bag). We then compared the performance of teen STOP-Bag and teen STOP-Bang. We found no significant differences between teen STOP-Bag and teen STOP-Bang. For all children, the AUC of teen STOP-Bag was 0.734 compared to the teen STOP-Bang AUC of 0.728 (p = 0.80).

## Discussion

In this community-based cohort of 312 children, the teen STOP-Bang instrument was found to have a high negative predictive value in assessing the likelihood of obstructive sleep apnea. The prevalence adjusted negative likelihood ratio is 0.05 (Table 2) which is consistent with an excellent negative predictive value. Given the sample prevalence of 8%, a teen STOP-Bang of less than 3 would reduce the odds of a child having sleep apnea from 1 in 13.5 to 1 in 263. Such a high negative predictive value makes the teen STOP-Bang a useful instrument to mitigate the need for obtaining a polysomnogram to further evaluate the presence of OSA. This may be of particular utility in children with learning problems or symptoms of attention-deficit hyperactivity disorder, as these symptoms may be related to sleep-disordered breathing in children [23, 26]. A low teen STOP-Bang score (less than 3) would suggest that OSA is unlikely. Additionally, a teen STOP-Bang score of 4 or greater did show increased specificity compared to a score of 3 (94% vs. 82–86%), suggesting that increasing teen STOP-Bang scores may be associated with an increased likelihood of OSA.

One previous study has evaluated the use of a modified STOP-Bang in children [27] and reported a sensitivity of 12% and specificity of 90% (negative predictive value of 0.67, positive predictive value of 0.37). There are several differences that likely explain differences between the study by Kadmon et al and our findings [27]. In the study by Kadmon et al, BMI and neck circumference cut-offs were absolute numbers and not age-adjusted percentiles. Additionally, elevated blood pressure was assessed by self-report, and not measured by American Heart Association guidelines as performed in our study. Also, we included the additional factor of academic problems as a question in substitution of age>50 that distinguishes our study from the prior study. Finally, the study by Kadmon et al included a wide age range (7–18 years old), whereas our data has shown that this tool is more accurate in older children when compared to younger children. The more adult-like phenotype of OSA in adolescents [28] may have contributed to the better test characteristics of the teen STOP-bang instrument in our study.

We found that the teen STOP-Bang instrument was more accurate in children who have progressed further through puberty. This is likely due to differences in the adolescent compared to pre-adolescent phenotype of OSA that more closely resembles that of adults, which is the population in which the STOP-Bang tool was originally developed [29]. Previous studies have shown that post-pubertal children show sex-related differences that are not seen in younger children [30]. A recent cohort study in older adolescents has also shown that risk factors for sleep-disordered breathing are different between younger children and adolescents [28].

An additional finding of our study was that while there was no significant difference in the results of parent report compared to teenage child report, there was a significant difference in pre-teen report compared to parent report. This finding may reflect that older adolescents may be more aware of their symptoms, or are more accurate reporters, although further research would be needed to definitively determine this. Prior research in this area, specifically in health-related quality of life, has shown conflicting results, with some studies showing good correlation between child self-report and parent-report [31, 32], while others have shown significant differences [33, 34].

Our study has several limitations. Our study included only Hispanic and Caucasian children by design, so the validity of these results in other ethnicities is unknown. There was a relatively small population (n = 25) of children with OSA in our study, validation of these results in a larger cohort would be useful. There were few children with moderate or severe OSA, which prevented analysis of teen STOP-Bang performance specifically in moderate-severe OSA, which has been shown to be of use in the adult STOP-Bang[35]. While there was a significant difference between ROC curves generated based on pubertal status, there was no difference in ROC curves by age. This is likely due to children undergoing puberty at varying ages, which results in a heterogeneous sample when age is used as a surrogate for pubertal status. However, age is likely a more implementable clinical measure than measuring SMR in children to screen for sleep-disordered breathing. Additionally, SMR was self-assessed based on a pictorial scale, and it is possible adolescents may not be accurate in their assessment, however, several studies have shown that adolescents can accurately measure pubertal development to within one sexual maturity rating stage of a trained examiner [36–38]. Finally, while increased neck circumference is a well-known risk factor for OSA in adults[39], and has been shown to be a predictive factor in children[40], there was not a significant difference in neck circumference seen in our population between children with and without sleep apnea. This may explain why the omission of neck circumference did not worsen the teen STOP-Bang test performance.

A major strength of this study was that it was conducted in a community-based cohort. Previous tools have been developed using children referred for polysomnograms, a population with a higher pre-test prevalence of OSA [8, 41]. The use of a community cohort improves the generalizability of our study to a primary care setting. Additionally, our study focused specifically on adolescent children; to our knowledge, there is no previously described tool for assessing the risk of OSA in this age group.

## Conclusions

We have developed a modification of the STOP-Bang tool for use in adolescent children. In community dwelling adolescents, the teen STOP-Bang questionnaire may be useful in stratifying the risk of OSA and determining the need for further evaluation for OSA and polysomnography. The implementation potential in general pediatric clinics, however, needs to be determined. Moreover, future prospective studies are needed to determine the validity of our teen STOP-Bang tool, particularly in other races and ethnicities which were not included in our study.

## Supporting Information

S1 Dataset TuCASA data utilized for the development of the teen STOP-Bang. (SAV)

## References

[1] Marcus CL, Brooks LJ, Draper KA, Gozal D, Halbower AC, Jones J, et al Diagnosis and management of childhood obstructive sleep apnea syndrome. Pediatrics. 2012;130(3):576–84. 10.1542/peds.2012-1671 . 22926173

[2] Kaemingk KL, Pasvogel AE, Goodwin JL, Mulvaney SA, Martinez F, Enright PL, et al Learning in children and sleep disordered breathing: findings of the Tucson Children's Assessment of Sleep Apnea (tuCASA) prospective cohort study. Journal of the International Neuropsychological Society: JINS. 2003;9(7):1016–26. 10.1017/S1355617703970056 . 14738283

[3] Friedman BC, Hendeles-Amitai A, Kozminsky E, Leiberman A, Friger M, Tarasiuk A, et al Adenotonsillectomy improves neurocognitive function in children with obstructive sleep apnea syndrome. Sleep. 2003;26(8):999–1005. . 14746381 10.1093/sleep/26.8.999

[4] Montgomery-Downs HE, Crabtree VM, Gozal D. Cognition, sleep and respiration in at-risk children treated for obstructive sleep apnoea. The European respiratory journal. 2005;25(2):336–42. 10.1183/09031936.05.00082904 . 15684300

[5] Tapia IE, Marcus CL. Newer treatment modalities for pediatric obstructive sleep apnea. Paediatric respiratory reviews. 2013;14(3):199–203. 10.1016/j.prrv.2012.05.006 . 23931720

[6] Erichsen D, Godoy C, Granse F, Axelsson J, Rubin D, Gozal D. Screening for sleep disorders in pediatric primary care: are we there yet? Clinical pediatrics. 2012;51(12):1125–9. 10.1177/0009922812464548 . 23203980

[7] Xu Z, Jiaqing A, Yuchuan L, Shen K. A case-control study of obstructive sleep apnea-hypopnea syndrome in obese and nonobese chinese children. Chest. 2008;133(3):684–9. 10.1378/chest.07-1611 . 18198258

[8] Chervin RD, Hedger K, Dillon JE, Pituch KJ. Pediatric sleep questionnaire (PSQ): validity and reliability of scales for sleep-disordered breathing, snoring, sleepiness, and behavioral problems. Sleep medicine. 2000;1(1):21–32. . 10733617 10.1016/s1389-9457(99)00009-x

[9] Constantin E, Tewfik TL, Brouillette RT. Can the OSA-18 quality-of-life questionnaire detect obstructive sleep apnea in children? Pediatrics. 2010;125(1):e162–8. 10.1542/peds.2009-0731 . 20026494

[10] Boynton G, Vahabzadeh A, Hammoud S, Ruzicka DL, Chervin RD. Validation of the STOP-BANG Questionnaire among Patients Referred for Suspected Obstructive Sleep Apnea. J Sleep Disord Treat Care. 2013;2(4). 10.4172/2325-9639.1000121 24800262 PMC4008971

[11] Chung F, Yegneswaran B, Liao P, Chung SA, Vairavanathan S, Islam S, et al STOP questionnaire: a tool to screen patients for obstructive sleep apnea. Anesthesiology. 2008;108(5):812–21. 10.1097/ALN.0b013e31816d83e4 . 18431116

[12] Goodwin JL, Enright PL, Kaemingk KL, Rosen GM, Morgan WJ, Fregosi RF, et al Feasibility of using unattended polysomnography in children for research—report of the Tucson Children's Assessment of Sleep Apnea study (TuCASA). Sleep. 2001;24(8):937–44. . 11766164 10.1093/sleep/24.8.937

[13] Rechtschaffen A KA. A manual of standardized terminology, techniques and scoring system for sleep stages of human subjects. NIH publication no 204 Bethesda, US Government Printing Office. 1968. doi: Bethesda, US Government Printing Office.

[14] Budhiraja R, Quan SF. Outcomes from the Tucson Children's Assessment of Sleep Apnea Study (TuCASA). Sleep medicine clinics. 2009;4(1):9–18. 10.1016/j.jsmc.2008.11.002 20161340 PMC2679504

[15] Witmans MB, Keens TG, Davidson Ward SL, Marcus CL. Obstructive hypopneas in children and adolescents: normal values. American journal of respiratory and critical care medicine. 2003;168(12):1540 10.1164/ajrccm.168.12.954 . 14668259

[16] Prevention CfDCa. Clinical Growth Charts. Available from: http://www.cdc.gov/growthcharts/clinical_charts.htm

[17] National High Blood Pressure Education Program Working Group on High Blood Pressure in C, Adolescents. The fourth report on the diagnosis, evaluation, and treatment of high blood pressure in children and adolescents. Pediatrics. 2004;114(2 Suppl 4th Report):555–76. . 15286277

[18] Lohman TG, Roche AF, Martorell R. Anthropometric standardization reference manual Abridged ed. Champaign, Ill.: Human Kinetics Books; 1991 vi, 90 p. p.

[19] Katz SL, Vaccani JP, Clarke J, Hoey L, Colley RC, Barrowman NJ. Creation of a reference dataset of neck sizes in children: standardizing a potential new tool for prediction of obesity-associated diseases? BMC pediatrics. 2014;14:159 Epub 2014/06/24. 10.1186/1471-2431-14-159 24952386 PMC4110068

[20] Taylor SJ, Whincup PH, Hindmarsh PC, Lampe F, Odoki K, Cook DG. Performance of a new pubertal self-assessment questionnaire: a preliminary study. Paediatric and perinatal epidemiology. 2001;15(1):88–94. . 11237120 10.1046/j.1365-3016.2001.00317.x

[21] Marshall WA, Tanner JM. Variations in the pattern of pubertal changes in boys. Archives of disease in childhood. 1970;45(239):13–23. 5440182 10.1136/adc.45.239.13 PMC2020414

[22] Marshall WA, Tanner JM. Variations in pattern of pubertal changes in girls. Archives of disease in childhood. 1969;44(235):291–303. 5785179 10.1136/adc.44.235.291 PMC2020314

[23] Goodwin JL, Kaemingk KL, Mulvaney SA, Morgan WJ, Quan SF. Clinical screening of school children for polysomnography to detect sleep-disordered breathing—the Tucson Children's Assessment of Sleep Apnea study (TuCASA). Journal of clinical sleep medicine: JCSM: official publication of the American Academy of Sleep Medicine. 2005;1(3):247–54. 16429591 10.5664/jcsm.26338 PMC1307497

[24] Raslich MA, Markert RJ, Stutes SA. Selecting and interpreting diagnostic tests. Biochem Medica. 2007;17(2):151–61. .

[25] Willis BH. Empirical evidence that disease prevalence may affect the performance of diagnostic tests with an implicit threshold: a cross-sectional study. BMJ Open. 2012;2(1):e000746 10.1136/bmjopen-2011-000746 22307105 PMC3274715

[26] Perfect MM, Archbold K, Goodwin JL, Levine-Donnerstein D, Quan SF. Risk of behavioral and adaptive functioning difficulties in youth with previous and current sleep disordered breathing. Sleep. 2013;36(4):517–25B. 10.5665/sleep.2536 23543901 PMC3595180

[27] Kadmon G, Chung SA, Shapiro CM. I'M SLEEPY: A short pediatric sleep apnea questionnaire. Int J Pediatr Otorhinolaryngol. 2014;78(12):2116–20. Epub 2014/10/12. 10.1016/j.ijporl.2014.09.018 . 25305064

[28] Spilsbury JC, Storfer-Isser A, Rosen CL, Redline S. Remission and Incidence of Obstructive Sleep Apnea from Middle Childhood to Late Adolescence. Sleep. 2014 . 25325456 10.5665/sleep.4318 PMC4262952

[29] Dayyat E, Kheirandish-Gozal L, Gozal D. Childhood Obstructive Sleep Apnea: One or Two Distinct Disease Entities? Sleep medicine clinics. 2007;2(3):433–44. 10.1016/j.jsmc.2007.05.004 18769509 PMC2084206

[30] Fuentes-Pradera MA, Sanchez-Armengol A, Capote-Gil F, Quintana-Gallego E, Carmona-Bernal C, Polo J, et al Effects of sex on sleep-disordered breathing in adolescents. The European respiratory journal. 2004;23(2):250–4. . 14979499 10.1183/09031936.03.00022003

[31] Sattoe JN, van Staa A, Moll HA, On Your Own Feet Research G. The proxy problem anatomized: child-parent disagreement in health related quality of life reports of chronically ill adolescents. Health and quality of life outcomes. 2012;10:10 10.1186/1477-7525-10-10 22276974 PMC3299605

[32] Theunissen NC, Vogels TG, Koopman HM, Verrips GH, Zwinderman KA, Verloove-Vanhorick SP, et al The proxy problem: child report versus parent report in health-related quality of life research. Quality of life research: an international journal of quality of life aspects of treatment, care and rehabilitation. 1998;7(5):387–97. . 9691719 10.1023/a:1008801802877

[33] Vetter TR, Bridgewater CL, McGwin G Jr. An observational study of patient versus parental perceptions of health-related quality of life in children and adolescents with a chronic pain condition: who should the clinician believe? Health and quality of life outcomes. 2012;10:85 10.1186/1477-7525-10-85 22824550 PMC3478968

[34] Baca CB, Vickrey BG, Hays RD, Vassar SD, Berg AT. Differences in child versus parent reports of the child's health-related quality of life in children with epilepsy and healthy siblings. Value in health: the journal of the International Society for Pharmacoeconomics and Outcomes Research. 2010;13(6):778–86. 10.1111/j.1524-4733.2010.00732.x 20561342 PMC3065295

[35] Chung F, Subramanyam R, Liao P, Sasaki E, Shapiro C, Sun Y. High STOP-Bang score indicates a high probability of obstructive sleep apnoea. Br J Anaesth. 2012;108(5):768–75. 10.1093/bja/aes022 22401881 PMC3325050

[36] Chan NP, Sung RY, Kong AP, Goggins WB, So HK, Nelson EA. Reliability of pubertal self-assessment in Hong Kong Chinese children. Journal of paediatrics and child health. 2008;44(6):353–8. 10.1111/j.1440-1754.2008.01311.x . 18476928

[37] Rollof L, Elfving M. Evaluation of self-assessment of pubertal maturation in boys and girls using drawings and orchidometer. Journal of pediatric endocrinology & metabolism: JPEM. 2012;25(1–2):125–9. 10.1515/jpem.2011.440 . 22570961

[38] Wu Y, Schreiber GB, Klementowicz V, Biro F, Wright D. Racial differences in accuracy of self-assessment of sexual maturation among young black and white girls. The Journal of adolescent health: official publication of the Society for Adolescent Medicine. 2001;28(3):197–203. . 11226842 10.1016/s1054-139x(00)00163-4

[39] Davies RJ, Ali NJ, Stradling JR. Neck circumference and other clinical features in the diagnosis of the obstructive sleep apnoea syndrome. Thorax. 1992;47(2):101–5. 1549815 10.1136/thx.47.2.101 PMC463582

[40] Katz S, Murto K, Barrowman N, Clarke J, Hoey L, Momoli F, et al Neck circumference percentile: A screening tool for pediatric obstructive sleep apnea. Pediatric pulmonology. 2015;50(2):196–201. 10.1002/ppul.23003 . 24574055

[41] Kadmon G, Shapiro CM, Chung SA, Gozal D. Validation of a pediatric obstructive sleep apnea screening tool. International journal of pediatric otorhinolaryngology. 2013;77(9):1461–4. 10.1016/j.ijporl.2013.06.009 . 23838544

